# p38 MAPK Signaling in Pemphigus: Implications for Skin Autoimmunity

**DOI:** 10.1155/2013/728529

**Published:** 2013-07-10

**Authors:** Athanasios Mavropoulos, Timoklia Orfanidou, Christos Liaskos, Daniel S. Smyk, Vassiliki Spyrou, Lazaros I. Sakkas, Eirini I. Rigopoulou, Dimitrios P. Bogdanos

**Affiliations:** ^1^Cellular Immunotherapy and Molecular Immunodiagnostics, Institute for Research and Technology-Thessaly (I.RE.TE.TH), 41222 Larissa, Greece; ^2^Institute of Liver Studies, Transplantation Immunology and Mucosal Biology, King's College London School of Medicine at King's College Hospital, Denmark Hill Campus, London SE5 9RS, UK; ^3^Department of Animal Production, Technological Educational Institute of Larissa, 41110 Larissa, Greece; ^4^Department of Medicine, Faculty of Medicine, School of Health Sciences, University of Thessaly, Viopolis, 41110 Larissa, Greece

## Abstract

p38 mitogen activated protein kinase (p38 MAPK) signaling plays a major role in the modulation of immune-mediated inflammatory responses and therefore has been linked with several autoimmune diseases. The extent of the involvement of p38 MAPK in the pathogenesis of autoimmune blistering diseases has started to emerge, but whether it pays a critical role is a matter of debate. The activity of p38 MAPK has been studied in great detail during the loss of keratinocyte cell-cell adhesions and the development of pemphigus vulgaris (PV) and pemphigus foliaceus (PF). These diseases are characterised by autoantibodies targeting desmogleins (Dsg). Whether autoantibody-antigen interactions can trigger signaling pathways (such as p38 MAPK) that are tightly linked to the secretion of inflammatory mediators which may perpetuate inflammation and tissue damage in pemphigus remains unclear. Yet, the ability of p38 MAPK inhibitors to block activation of the proapoptotic proteinase caspase-3 suggests that the induction of apoptosis may be a consequence of p38 MAPK activation during acantholysis in PV. This review discusses the current evidence for the role of p38 MAPK in the pathogenesis of pemphigus. We will also present data relating to the targeting of these cascades as a means of therapeutic intervention.

## 1. Introduction

The skin represents the first organ of the body exposed to the external environment and thus serves as the primary barrier of the immune defense system. Its key role is to maintain protection against hazardous environmental threats such as microorganisms and viruses [1, 2]. The epidermis is the outmost cellular tissue of the skin and expresses several proteins that orchestrate the essential protective functions. Inflammatory mediators such as prostaglandins, histamines, cytokines, and chemokines are synthesized and secreted from keratinocytes regulating the skin's immune responses [3, 4]. 

When this epithelial barrier is compromised due to the deterioration of skin tissue integrity, patients are at high risk of fluid and electrolyte loss, as well as infection. If left untreated, conditions can be fatal. Even though the mechanisms of skin damage are essentially the same as the ones that control protective immunity and despite the evolution of sophisticated anti-inflammatory and tissue repair mechanisms, the formation of immune complexes against self-antigens (a hallmark of autoimmunity) initiates a sustained inflammatory response characterized by autoreactive immune cells, cytokines, and autoantibody production [5–8].

Pemphigus signifies a distinctive skin-specific acquired autoimmune disease characterized by intraepidermal blistering, which is induced by autoantibodies against desmosomal cadherins, desmoglein 1 (Dsg1), and Dsg3 [9–11]. Three typical variations are known and are classified as pemphigus vulgaris (PV), pemphigus foliaceus (PF), and some other variants distinguished by the degree of inflammation, the level of separation in the epidermis, and immunologic properties of autoantigens. PV-IgG-targeted cells and triggered signaling pathways are tightly linked to the secretion of cytokines and chemokines that initiate and perpetuate inflammation and subsequent tissue damage [12–14]. 

This review will discuss up to date evidence of the role of certain key signaling pathways such as p38 MAPK in the pathogenesis of pemphigus, with particular emphasis on the protagonist cells. These data may help us to better understand the signaling cascade pathways of other distinct immune-mediated skin pathologies such as psoriasis [15–18].

## 2. Pemphigus Vulgaris: An Ideal Model to Study Autoimmunity

PV is a potentially lethal autoimmune blistering disease that affects the skin and mucous membranes [23]. It is a relatively rare disease with an incidence of about 1–3.5 cases per 100,000 individuals annually worldwide, being more frequent amongst middle aged individuals. Despite its low frequency, research on PV has benefited from some distinct advantages fundamental for the elucidation of its molecular pathogenesis and the development of unique targeted therapies [24, 25]. Firstly, a distinct clinical pathology is easily observable; secondly, the molecular targets (autoantigens and reactive autoantibodies) are clearly defined; thirdly, some successful *in vivo* models are readily available; and lastly but not the least, the skin is easily accessible to topical and systemically delivered pharmaceutics. Detection of tissue-specific and serum autoantibodies and further characterization of their molecular specificity are mandatory for the diagnosis of autoimmune blistering diseases. For this purpose, various specific immunoassays, including immunofluoresence, enzyme-linked immunosorbent assay and immunoprecipitation, have been developed [26–28]. 

The trademark of PV histopathology is intraepidermal blistering-associated acantholysis. Acantholysis is defined as the loss of adherence between epithelial cells and structural components maintaining cell-cell and cell matrix adhesion in the skin and mucous membranes [29]. The junctions that facilitate cohesiveness between epithelial cells are termed desmosomes. Desmosomes are principally located in tissues that experience mechanical forces such as the skin and heart and function as anchor sites for cytoskeleton microfilaments [30]. The desmosomal proteins responsible for mediating cellular adhesion are called cadherins and include the Dsg and desmocollins (Dsc). These constitute the protein components of desmosomes that are responsible for mediating cellular adhesion [31]. In the epidermis, a total of four Dsg (Dsg1–4) and three Dsc (Dsc1–3) isoforms are expressed. Autoantibodies directed against the Dsgs are typical in PV, and several studies have demonstrated that antibodies directed against Dsg1 and Dsg3 induce acantholysis within the epidermis and mucous membranes [32–34]. In contrast, PF is characterized by antibodies directed against Dsg1 only [35]. Seminal studies by the group of Jensen and Lazarus have suggested that keratinocyte inflammatory responses are probably involved in PV-IgG-induced acantholysis [36, 37], but the direct link with Dsgs was not addressed at that time.

The expression of Dsg3 is mainly confined to stratified epithelia. In the epidermis, it is expressed throughout the basal and the spinous layers. Dsg3 inactivation is sufficient to induce the characteristic blistering pathology of PV patients [38, 39]. Mice injected with Dsg3-specific IgG purified from PV patients have been shown to develop acantholysis. Similarly, genetic deletion of Dsg3 or Dsc3 in mice results in a phenotype resembling PV in its mucosal-dominant form [40–42]. Dsg3-deficient littermates suffer from oral erosions and epidermal blistering in areas subjected to extensive mechanical stress. Recombinant Dsg3 can also be used to deplete patient sera of disease-causing antibodies, demonstrating that autoantibodies are indeed directed against Dsg3 and induce blistering. The exact mechanism of this histopathology is defined loss of keratinocyte adhesions by binding of anti-Dsg3 antibodies to Dsg3 through steric hindrance, internalization of Dsg3, changes in molecular integrity, and subsequent intracellular signal transduction [43].

Prevention of Dsg3 endocytosis and/or inhibition of downstream signaling pathways can prevent PV autoantibody-induced loss of adhesion in both cell culture and animal model systems [21]. Tyrosine kinase initiated pathways, protein kinase C (PKC), RhoA, and c-myc have all been implicated in the series of events leading to loss of adhesion in keratinocytes treated with PV IgG [44, 45]. Particularly convincing data has recently been published regarding the involvement of the p38 mitogen-activated protein kinase (MAPK) pathway, which has been linked to both Dsg3 endocytosis and the loss of keratinocyte adhesion in response to PV IgG [19, 21, 46–48]. Interestingly, the pathogenic activity of polyclonal PV IgG can be attributed to p38-MAPK-dependent clustering and endocytosis of Dsg3, whereas pathogenic monoclonal Dsg3 antibodies can function independently of this pathway [49]. Detailed evidence is beginning to accumulate that activation of signaling molecules may have an important role in the ability of pathogenic pemphigus IgGs to induce blistering. In fact, both p38 and downstream mediators such as heat shock protein (HSP) 27 may be part of this important process [19].

## 3. p38 MAPK Pathway Activation, Detection, and Relevance to Skin Autoimmunity

The p38 MAPK signaling pathway is a critical participator in the regulation of cellular and humoral autoimmune responses [50, 51]. Usually initiated by cellular stresses or inflammatory cytokines, its main task is to orchestrate cytokine gene expression including tumor necrosis factor (TNF)-*α* and interferon-*γ* (IFN-*γ*) by means of transcriptional and posttranscriptional mechanisms such as stabilization of mRNA transcripts [52–56]. P38 MAPK is also important for cellular survival, proliferation, differentiation, and apoptosis [57]. An increasing number of studies have provided data demonstrating the significant role of this cascade in the pathogenesis of several immune-mediated diseases, including rheumatoid arthritis (RA), Sjögren's syndrome, systemic lupus erythematosus (SLE), autoimmune hepatitis (AIH), and psoriasis to name a few [58]. Defects in p38 MAPK pathway can explain the increased expression of proinflammatory cytokines seen in psoriasis [59]. For instance, there is increased TNF-*α* protein expression, but similar mRNA levels, in lesional compared with nonlesional psoriatic skin, demonstrating that TNF-*α* expression in psoriatic skin is regulated posttranscriptionally by p38 MAPK, and therefore p38 signaling pathway can be targeted therapeutically [60–62].

The activity of p38 MAPK can be studied in available experimental models and clinical material such as peripheral blood mononuclear cells (PBMCs) and pathological tissue from patients with autoimmune diseases [63]. The conventional techniques for p38 detection include western blotting, immunoprecipitation, and confocal immunofluorescence microscopy that detects the active p38 kinase translocated to the cell nucleus. However, these applications require large populations of homogenous cells, and the data obtained are not fully quantitative. Extended culturing of primary cells is also mandatory in order to maximize cell densities. It should be noted that extensive culturing periods, often in the presence of Interleukin (IL)-2 and other growth factors, can affect or bias signaling analyses that depend on interaction of cell receptors and phosphorylation of intracellular proteins.

Optimized protocols based on sensitive phospho flow cytometry has been recognised as promising alternatives for the investigation of the phosphorylation of p38 MAPK within different PBMCs [64–66]. Phospho-specific flow cytometry technology may help us to better understand the enigmatic role of this signalling cascade in the induction of autoimmunity, as well as its role in immunosuppressive-induced remission [67]. Its main advantage is that it delivers extremely rapid, sensitive, and fully quantifiable observations. Moreover, it allows multiparametric analysis of samples containing mixed subpopulations such as PBMCs. This is feasible upon successful combination of fluorochrome conjugated antibodies against surface markers such as anti-CD3 and anti-CD56 and intracellular epitopes such as phospho-p38 [63]. Therefore, direct analysis of rare populations becomes feasible, as does multiparametric detection of several epitopes within these cells. We have optimized methodology for the successful application of phosphor-specific flow cytometry in order to detect phosphorylated p38 MAPK within innate immune cells such as NK and NKT [63]. In addition, we have provided technical instructions permitting simultaneous flow cytometric measurement of p38 MAPK phosphorylation and intracellular cytokine production. This might be of special value in cases of autoimmunity or cancer where the unavailability of numerous cells from immune-compromised individuals hampers analysis of their biological responses.

Given the key role p38 MAPK signaling pathway plays in inflammatory responses through the production of cytokines and inflammatory mediators, its inhibition is considered to be a promising target for chronic inflammatory diseases [50, 68]. Several pharmaceutical companies have invested heavily on the development of agents specifically inhibiting p38 MAPK activation. An increasing number of novel p38 MAPK inhibitors have been used in experimental studies and clinical trials and have helped us to further define the role of p38 MAPK [69–71]. For instance the *α*-selective p38 MAPK inhibitor, SCIO-469, acts as a topical anti-inflammatory agent via the p38 MAPK pathway to reduce neutrophil-induced acute inflammation in the skin, and observations in clinical models suggest that selective p38 MAPK inhibition may be an effective therapeutic strategy to manage acute skin inflammation [72].

## 4. p38 MAPK and Pemphigus Vulgaris

The p38 MAPK signaling pathway participates in chronic inflammatory skin pathologies, as p38 MAPK inhibitors reduce skin inflammation in various rodent models of human skin diseases [73]. For example, p38 MAPK inhibitors protect the epidermis against the acute damaging effects of ultraviolet irradiation by blocking apoptosis [74]. In addition, topical p38 MAPK inhibition reduces dermal inflammation and epithelial apoptosis in burn wounds [75, 76]. 

**Figure 1:**
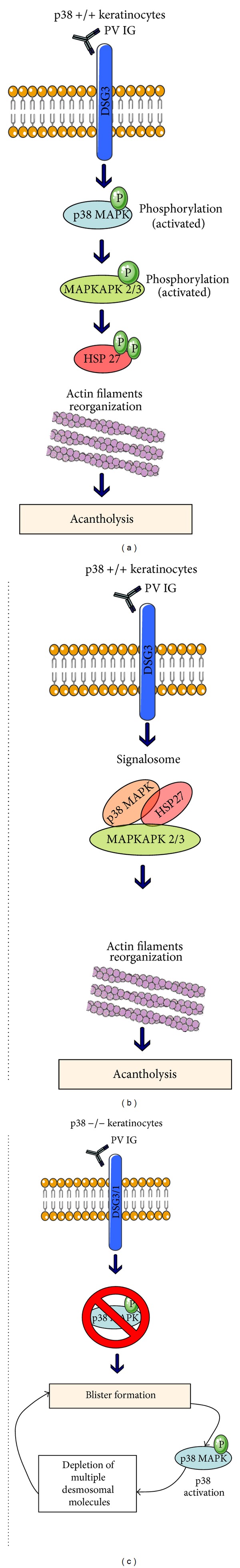
The role of p38 MAPK in the induction of pemphigus vulgaris (PV). There are at least three potential p38-MAPK-related mechanisms involved in the pathogenesis and/or the progression of PV. (a) The binding of pathogenic autoantibodies targeting Dsg3 in keratinocytes initiates an array of signals leading to the activation of p38 MAPK cascade with subsequent phosphorylation of MAPKAPK (mitogen-activated protein kinase-activated protein kinases 2/3) and heat shock protein 27 (Hsp 27). The final outcome of these events is actin filaments reorganization and induction of acantholysis [19]; (b) p38 MAPK, MAPKAPK, and Hsp27 may form a complex (signalosome) that regulates the reorganization of actin filaments and the induction of acantholysis [20]; (c) studies in p38−/− keratinocytes demonstrate a p38 MAPK-independent blister formation. The subsequent activation of this pathway, however, can lead to *de novo* depletion of multiple desmosomal molecules, further facilitating spontaneous blister formation [21]. This latter hypothesis indicates that p38 MAPK signaling may not be responsible for the induction of PV but could play a role for the progression of the disease.

What is of particular importance is the finding of an autoantibody-mediated autoimmune skin disease where p38 MAPK is directly activated by the same autoantibodies and implicated in disease pathogenesis [12]. This occurs in PV and (regarding p38 MAPK) David Rubenstein's lab has demonstrated that pemphigus-IgG binding to keratinocytes augmented intracellular phosphorylation events [13, 19]. In their studies, cultured human keratinocytes with IgG purified from patients with PV activate the p38 MAPK and lead to phosphorylation of the small HSP 27, actin cytoskeleton reorganization, as well as the collapse of the intermediate actin microfilaments. In isolated tissue cultures where p38 MAPK was inhibited, the phosphorylation of HSP 27 and cytoskeleton reorganization was greatly diminished. The same group of investigators have generated a mouse model in which PV was passively transferred. Inhibiting p38 MAPK in these mice prevented the formation of blisters *in vivo*. It also appears that inhibiting p38 MAPK blocks the pathogenic IgG from inducing blistering in the skin in mouse models of PV. Importantly, p38 MAPK and HSP27 phosphorylation has also been observed in the epidermis surrounding the skin lesions of both psoriasis and PV [77, 78] (Figure 1).

Timing of p38 MAPK activation is critical for understanding the hierarchy of signaling events leading to acantholysis. Time course experiments demonstrated that the activities of Src and EGFRK peak at 30–60 min after exposure to PV IgG suggesting that engagement of Src/EGFRK is a critical step that generates signals from ligated antigens to the intracellular effectors such as p38 affecting keratinocyte adhesion and viability [46]. Phosphorylated p38 MAPK by antibodies from PV patients can be detected as early as 15 min; however, the majority of PV IgGs induced peak p38 activity after a prolonged incubation. In cultured keratinocytes, p38 knockdown abrogated desmosomal Dsg3 reduction by PV mAbs, whereas exogenous p38 activation caused internalization of Dsg3, Dsc3, and desmoplakin [21]. It was therefore suggested that p38 MAPK may not be essential for the loss of intercellular adhesion in PV but may function downstream to augment blistering via Dsg3 endocytosis.

Additionally, Marchencko et al. have demonstrated the circulation of antimitochondrial antibodies against various poorly defined mitochondrial autoantigens in sera from PV patients [47]. These autoantibodies appear to have the ability to penetrate keratinocytes and react with mitochondrial proteins. Data provided in the same study suggested that downstream signaling of antimitochondrial antibodies involves JNK and late p38 MAPK activation, further underlying the important role for these signaling cascades in non-Dsg3 or Dsg1 autoantibody-specific autoimmune blistering. 

Whether pemphigus-IgG-augmented p38 signaling is directly or indirectly linked to the pathogenesis of pemphigus through the secretion of cytokines and chemokines that initiate or activate inflammatory events remains debatable [79, 80]. Natural killer (NK) cells are professionally programmed to induce rapid bursts of cytokines and chemokines during innate immune responses [81]. Recent studies on innate immunity have noticeably expanded our understanding of the function of NK cells in health and disease [82]. Besides their well-known functions in cancer and autoimmunity, significant contributions from NK cells to allergies, and various skin diseases have emerged. NK cells play an important part in skin autoimmunity, such as in psoriasis [83]. Evidence that NK may also participate in the pathobiology of pemphigus is beginning to emerge. Takahashi et al. showed that a higher percentage and number of NK cells are present in the peripheral blood of PV patients [84]. These expressed the CD69 cell surface marker indicating that the NK cells were activated and expanded in the peripheral blood of PV patients. They also exhibited lower levels of perforin and granzyme defects in their cytotoxic ability and increased levels of Interleukin 10 expression.

**Figure 2:**
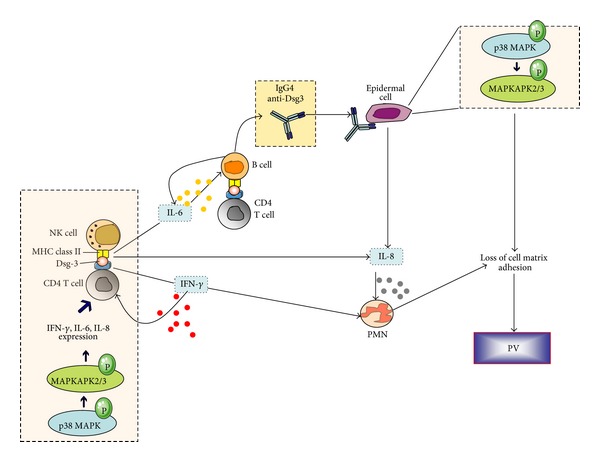
Schematic representation of the suggested interplay between natural killer (NK), CD4+ T, B cells, and polymorphonuclear cells (PMN) during pemphigus pathogenesis [22]. NK cells accumulate from the bloodstream to the epidermis and act as antigen-presenting cells by introducing desmoglein (Dsg3) peptides to CD4+ T cells. p38 MAPK phosphorylation within lymphoid subpopulations such as NK and CD4+ T cells induces expression of IFN-*γ*, IL-6, and IL-8 that amplify the inflammatory response, MHC-II presentation, and autoantibody production by B cells. The activated T cells further enhance B-cell anti-Dsg3 secretion and presentation to epidermal cells. Anti-Dsg3 activates the p38 MAPK pathway within keratinocytes leading to MAPK-activated protein kinase 2 (MK-2) mediated heat shock protein 27 (Hsp27) phosphorylation, actin microfilament reorganization, and acantholysis.

 In another study by Stern et al., CD4+ T cells from the PBMCs and perilesional skin of PV patients were cocultured with CD56+ CD3-NK cells from the PBMCs of the same patients [22]. In the presence of Dsg3 peptides, CD4+ T cells proliferated, indicating that NK cells functioned as antigen-presenting cells. Supernatants from these cocultures and serum from the same patients with active PV had statistically significantly elevated levels of IFN-*γ*, IL-6, and IL-8, compared with controls indicating that the NK cells stimulated CD4+ T cells to produce proinflammatory cytokines in a similar manner to other autoimmune pathologies [85]. These data have led to the formulation of a hypothetical scenario for the p38 MAPK-induced pathogenesis of PV (Figure 2). In the microenvironment of the affected tissues, NK cells may present Dsg3 peptides to resident and circulating CD4+ T cells which proliferate and produce various cytokines. Both Dsg3-reactive Th1 and Th2 cells have been detected at similar frequencies in studied PV patients, yet the numbers of autoreactive Th1 cells exceeded those of Th2 cells in chronic active PV. The *in-vivo*-activated NK cells may also travel to lymph nodes, spleen, and bone marrow and stimulate B cells to produce high levels of pathogenic autoantibodies [8, 86]. 

NK cells are controlled by activating and inhibiting receptors. The killer-cell immunoglobulin-like receptors (KIRs) and their cognate HLA class I ligands are crucial for NK regulation. Activating KIR genes have been utilized in risk estimation for autoimmunity [87]. In a recent report, activating KIR and HLA Bw4 ligands have been demonstrated to be associated with decreased susceptibility to PF [88]. Activation of NK cells appears to be critical for the clinical manifestations and subsequent clinical stages of autoimmune blistering diseases. Based on the latest observations in the literature, a vital role of NK cells in the pathogenesis of PV is brought to light. However, more research is necessary in order to delineate the precise molecular mechanisms and activated signaling pathways in NK cells. There is currently no thorough information to the activation of p38 MAPK in NK and NKT cells from PF and PV patients.

## 5. Targeting p38 MAPK in Pemphigus: Corticosteroids versus Monoclonal Antibodies

The mainstay of PV treatment is systemic administration of corticosteroids aiming at reducing inflammation and autoantibody production [89, 90]. PV patients have traditionally been treated with glucocorticoids and adjuvant immunosuppressive therapies. However, clinical remission has been achieved in only 30% of patients. Methotrexate, mycophenolate mofetil, or cyclophosphamide are typically introduced as steroid sparing agents, since many patients experience severe side effects from glucocorticoid-induced immunosuppression [91, 92]. Newer modalities of treatment, such as B-cell depletion therapy with rituximab, have begun to show some promise in such patients. Rituximab is increasingly used in patients with PV with inadequate response to conventional therapy [93, 94].

Corticosteroids dampen inflammatory responses, and this occurs at least in part by inducing rapid and prolonged expression of MAP kinase phosphatase 1 (MKP-1), which potently inactivates p38 MAPK [95]. Any attempt for discussing therapeutic inhibition of p38 MAPK pathway stems from the fact that natural negative feedback mechanisms exist to guarantee that, within cells, MAPKs are not activated *ad infinitum*. In this regard, MAP kinases can themselves induce different types of protein phosphatases that dephosphorylate and cease their function. These are termed dual-specificity phosphatases (DUSPs), and each member of the DUSP family has a unique set of properties including tissue distribution, subcellular localization, and precise substrate affinity and specificity [96]. For example, MKP-1 targets primarily p38 MAPK contrary to MKP-2 which preferentially dephosphorylates ERK and JNK [97–99]. The strength and duration of p38 MAPK activation are often the critical determinant of cellular responses regulated by the action of these phosphatases [100]. In psoriatic lesions the p38 MAPK negative feedback mechanism provided by DUSP1 seems to be defective. DUSP1 mRNA expression was demonstrated to be significantly downregulated in psoriatic skin lesions as compared with paired samples of nonlesional psoriatic skin [101]. Downregulation of DUSP1 may contribute to the sustained inflammatory response seen in psoriasis. However, there is no information available regarding the expression of MKP-1 in PV. Yet, inhibition of p38 MAPK by MKP-1-dependent mechanisms leads to downregulation of IL-8, one of the cytokines implicated in the pathogenesis of PV [22, 56, 98].

As previously mentioned, p38 MAPK inhibition prevented blistering in the murine model of PV. Early clinical studies focusing on targeting p38 MAPK in inflammatory disorders, such as rheumatoid arthritis, Crohn's disease, and psoriasis, raised significant safety concerns [70]. A novel allosteric p38 MAPK inhibitor, KC-706 (Kémia Inc), has been tested during a phase II multicenter, open-label trial in patients with active PV [102]. The safety and efficacy of KC-706 in accomplishing remission, while maintaining stable doses of corticosteroids, were monitored over a 3-month period. KC-706 had been administered orally to 15 patients with PV. Approximately half of the patients exhibited a partial response to treatment, while the remaining patients either failed to improve or deteriorated [102]. Disappointingly, the inhibitor had to be abandoned due to severe adverse reactions. Thus the notion that p38 MAPK signaling is directly involved in the pathogenesis of PV, and hence p38 MAPK inhibitors can be ideal treatment agents needed to be treated with caution. Nevertheless, there is a continuous generation of biologics targeting the p38 pathway in a more sophisticated manner aiming to avoid undesirable side reactions [14, 24, 48, 103].

 Currently, there is an advent of certain other biologics and monoclonal antibodies that have shown particular potential in the treatment of PV. For example rituximab is a chimeric monoclonal antibody that targets the CD20 molecule on B cells resulting in their depletion [104]. Administration of rituximab has been approved for certain lymphomas, RA, chronic lymphocytic leukemia, and Wegener's granulomatosis [105, 106]. It is also used in certain cases to treat PV [107–109]. The rationale for the use of rituximab in patients with PV is based on its ability to deplete CD20+ B cells that presumably produce pathogenic antibodies. Antibodies against CD20 can activate complement and induce antibody-dependent cellular cytotoxicity (ADCC) in B lymphocytes. In B lymphocytic leukemia cells, cross-linking of rituximab induced strong and sustained phosphorylation of p38 MAPK [104]. Introduction of the p38 inhibitor completely blocked signaling downstream of p38, which was evident by the absence of MK2 activity and significantly reduced the degree of anti-CD20-induced apoptosis. Therefore the chimeric anti-CD20 antibody rituximab induces apoptosis in B-cell chronic lymphocytic leukemia cells through a p38-mitogen-activated protein-kinase-dependent mechanism. Yet, in 2F7 non-Hodgkin's lymphoma cells, rituximab inhibited p38 MAPK and NF-kappaB activity and downregulated IL-10 expression via Sp1 [110]. Interestingly, other monoclonal antibodies such as the anti-TNF monoclonal antibody adalimumab (Humira) rapidly inhibit p38 MAPK activity in lesional psoriatic skin preceding clinical improvement [62, 101]. The activities of ERK1/2, MSK1/2, and MK2 and the levels of TNF-*α* were also reduced. The clinical benefits of anti-TNF antibodies adalimumab (Humira), etanercept (Enbrel), and infliximab (Remicade) in the treatment of PV have been previously reported [111, 112]. 

## 6. Concluding Remarks

It has become apparent that p38 MAPK is involved in skin autoimmune diseases such as pemphigus. Nevertheless, its targeting can lead to completely unpredictable outcomes and certain adverse side effects depending on the different cells participating in skin autoimmune pathogenesis. PV pathogenesis is still disputed, and treatment remains perplexing [113].

Activating p38 MAPK in autoreactive B cells will enhance their apoptosis and cessation of autoantibody production. As for keratinocytes, the *de facto* inflicted cell type in PV, a different strategy might be necessary since apoptosis and acantholysis appear to be tightly linked to each other. The time course of p38 MAPK activation, as well as the ability of inhibitors of p38 MAPK to block activation of the proapoptotic proteinase caspase-3, suggests that induction of apoptosis is a consequence of p38 MAPK activation during acantholysis in PV [13]. Therefore, acantholysis can be prevented by inhibitors of p38 MAPK signaling kinases, in addition to other known targets such as the mammalian target of rapamycin, Src, EGFR kinase, phospholipase C, calmodulin, and protein kinase C, as well as inhibitors of executioner caspases [114]. 

In PV, skin infiltrating NK, NKT, and T cells, prolonged p38 MAPK activity will favor an uncontrolled self-perpetuating inflammatory loop and generation of auto-reactivity. If we are to induce apoptosis of autoreactive T cells, it may be wise to spare their assassins, namely, NK and NKTs. Therefore, careful monitoring of p38 signaling events within each cell type is of paramount importance. The advent of phospho-specific flow cytometry technology allows multiparametric analysis of rare populations and multiparametric detection of several epitopes within those cells. A typical example of how powerful this technology can become is the analysis of p38 phosphorylation within rare human CD3+ CD56+ cells [63]. We are currently capable of detecting p38/IFN-*γ* double positive NK and NKT cells using optimized phospho-flow cytometry protocols. This has been tested in cells from healthy individuals and in peripheral blood NK and NKT cells from patients with AIH and primary biliary cirrhosis (PBC) [115–119]. In AIH, p38 MAPK pathway is activated in the NKT cells, and the magnitude of this activation parallels the disease activity status of the patient [67]. Phospho-p38 MAPK positive NKT cells were more frequent in patients tested at diagnosis than in patients with immunosuppressive drug-induced remission.

 The holy grail of current research in PV and PF is to be able to achieve and maintain clinical remission without extensive use of corticosteroids. This is crucial since glucocorticosteroids can only block but not reverse acantholysis. Since p38 MAPK is phosphorylated in PV and is one of the targeted molecules by glucocorticoids, advancing our knowledge on the exact kinase phosphorylation/dephosphorylation events in every single cell involved can provide fresh impetus for further research into the precise role of this enigmatic signaling cascade in pemphigus and other autoimmune skin diseases. This will greatly facilitate to resolve debates on autoimmune molecular pathogenesis and open novel perspectives on successful targeted therapies.

## Abbreviations

AIH: Autoimmune hepatitis
Dsc: Desmocollin
Dsg: Desmoglein
DUSP: Dual specificity phosphatase
HSP: Heat shock protein
IFN: Interferon
IL: Interleukin
LPS: Liposaccharide
MAPK: Mitogen activated protein kinase
PF: Pemphigus foliaceus
PV: Pemphigus vulgaris
RA: Rheumatoid arthritis
Th1: T-helper 1
TNF: Tumor necrosis factor.
